# Construction Notes

**Published:** 1905-08-05

**Authors:** 


### CONSTRUCTION NOTES.

Mrs. Thomas Wharrie has given the munificent donation of £5,000 to St. Mary's Hospital.

The late Miss Agnes Abercrombie has bequeathed £1,000 to the Edinburgh Royal Infirmary.

The late Miss Frances Maria Wilbraham, of Chester, has left the income of her estate, valued at upwards of £8,000, and subject to her sister's life interest, to the Chester Infirmary, as well as the sum of £250 to the same institution.

The late Miss Clara Petter, of Bournemouth, has bequeathed £1,000 each to the Zenana Bible and Medical Mission and Hahnemann Convalescent Hospital, Bournemouth, and £500 each to the Royal Victoria Hospital, Bournemouth, and the North Devon Infirmary, Barnstaple, and £300 to the Royal Hospital for Incurables. The late Mr. Richard Taylor, of Leicester, bequeathed £1,000 to the Leicester Provident Dispensary.

The late Mrs. Elizabeth Eleanor O'Neill, of Southmere, Woodberry Down, left the ultimate residue of her estate, valued at £9,281, subject to her husband's life interest and other provisions, to be equally divided between Guy's Hospital, the Great Northern Central Hospital, the Brompton Hospital for Consumption and Diseases of the Chest, and the Royal National Hospital for Consumption and Diseases of the Chest at Ventnor.

A site for a sanatorium under the auspices of the National Committee for the establishment of sanatoria, has been secured at Benenden in Kent. It is intended to erect an institution for 200, men and women, at a cost of £50,000. We are glad to learn that the intention is to provide an inexpensive class of building. The Post Office employés are to benefit by having secured to them a certain number of beds, towards the maintenance of which they will contribute.

